# Phenolic characterization and nutraceutical evaluation of by‐products from different globe artichoke cultivars

**DOI:** 10.1002/jsfa.14232

**Published:** 2025-03-22

**Authors:** Giusy Rita Caponio, Mirco Vacca, Laura Scalvenzi, Alessandro Annunziato, Roccangelo Silletti, Claudia Ruta, Graziana Difonzo, Maria De Angelis, Giuseppe De Mastro

**Affiliations:** ^1^ Department of Bioscience, Biotechnology and Environment University of Bari Aldo Moro Bari Italy; ^2^ Department of Soil, Plant and Food Sciences University of Bari Aldo Moro Bari Italy

**Keywords:** artichoke, by‐product valorization, sustainability, bioactive compounds, polyphenols, prebiotic activity

## Abstract

**BACKGROUND:**

The globe artichoke (*Cynara cardunculus* var. *scolymus* L.) is extensively cultivated in the Mediterranean region, with Italy being a leading producer. Industrial processing of artichoke plants generates substantial amounts of residual materials, which are discarded annually. This accumulation of biowaste presents environmental challenges. However, these by‐products remain rich in phytochemicals, such as dietary fibers, phenolic acids, sesquiterpene lactones, flavonoids, vitamins and minerals, similar to those found in the edible parts of the plant. The present study aimed to evaluate the potential of artichoke by‐products by analyzing aqueous extracts (AEs) from seven cultivars, comprising two commercial hybrids and five local varieties, focusing on their phenolic content, antioxidant activity, and prebiotic potential.

**RESULTS:**

The primary phenolic compounds identified in the AEs were hydroxycinnamates, notably 5‐*O*‐caffeoylquinic acid and 3,5‐di‐*O*‐caffeoylquinic acid, and flavonoids, primarily apigenin‐7‐*O*‐rutinoside, luteolin and luteolin‐7‐*O*‐rutinoside. These bioactive compounds were more abundant in two of the five local varieties, with concentrations exceeding those in commercial hybrids by more than twofold. Local varieties consistently exhibited higher total phenolic content and greater antioxidant activity, as determined by the DPPH (i.e. 2,2‐diphenyl‐1‐picrilyhydrazil) assay. Furthermore, local varieties demonstrated a remarkable prebiotic potential, supporting more robust probiotic cell growth and resulting in greater acidification compared to commercial hybrids.

**CONCLUSION:**

The findings highlight the potential for valorizing artichoke biowastes as dietary supplements. The rich functional and bioactive properties of these by‐products, particularly in local varieties, offer promising applications in food‐related industries. This approach not only leverages their nutritional benefits, but also addresses environmental concerns by valorizing biowaste. © 2025 The Author(s). *Journal of the Science of Food and Agriculture* published by John Wiley & Sons Ltd on behalf of Society of Chemical Industry.

## INTRODUCTION

The globe artichoke (*Cynara cardunculus* L. var. *scolymus* (L.) Fiori) is a herbaceous perennial plant from the *Asteraceae* family, native to the Mediterranean area.^1^, ^2^ This vegetable is highly valued in Mediterranean cuisine and has been introduced to other regions worldwide. Currently, the annual global production of artichoke heads is approximately 1320 kt, with significant cultivation in Southern Europe (Italy, Spain, France, Greece), the Middle East (Turkey, Syria, Israel), North Africa (Egypt, Morocco, Algeria, Tunisia), South America (Peru, Argentina, Chile), the USA and, recently, in China.^3^ Italy is the leading producer, generating around 475 kt per year, and boasts the greatest biodiversity of artichokes characterized by numerous local clonal varieties with high heterozygosity as a result of genetic instability in early genotypes and inherent heterogeneity in propagation.^4^ Of the globe artichoke, the germplasm includes approximately 100–120 genetically distinct genotypes.^5^ Although categorized by harvest time (early or late) and morphological traits, such as bract color and presence of spines, it was previously suggested a classification of the globe artichoke germplasm into four groups, in detail, *Spinosi*, *Violetti*, *Romaneschi* and *Catanesi*.^6^ However, among overall this heterogenicity, only 11–12 genotypes hold major commercial significance.^5^

The edible portion of this plant is the immature flower head, known as the *capitulum* or head, which is composed of a receptacle surrounded by fleshy bracts. The receptacle is fully edible and is enjoyed globally in various forms such as raw, boiled, steamed and fried, and as an ingredient in numerous recipes because its pleasant sensory qualities and health benefits. Both the vegetative and reproductive parts of the globe artichoke are rich in phytochemicals, including inulin, minerals, vitamin C, inositol, sesquiterpene lactones and polyphenolic compounds.^4^, ^7^ Because of this composition, artichokes are highly regarded for their nutraceutical and medicinal properties.^8^ Various studies have focused on the antioxidant properties featuring artichoke and beneficial effects on liver health, which appear to be closely linked to the polyphenolic content, particularly featured by mono‐ and di‐caffeoylquinic acids and flavonoids.^9^ It is important to note that leaves, external bracts and stems are not suitable for human consumption and, therefore, are considered organic waste generated during the industrial processing of artichoke. Regrettably, the inedible part of the artichoke, accounting between 70 and 85% of the plant biomass,^10^ results in the loss of essential phytochemical substances found in different parts of the plant including in by‐products.

At present, the pivotal role of the circular and green economy is considered as the best driver ‘to adapt or transform the current economy towards a more sustainable one’^11^ and, in line with this, research is working with the purpose of characterize residual bioactive compounds from by‐products, including those of artichoke, to support the human wellbeing and health by diet.^12^, ^13^, ^14^ Previous studies have investigated the polyphenol profile of globe artichoke cultivars, exploring variations influenced by genotype,^15^, ^16^, ^17^, ^18^, ^19^ differences in molecular accumulation across plant parts^20^, ^21^ and the impact of different agronomic practices.^22^, ^23^, ^24^ However, few studies have specifically examined polyphenol recovery from waste biomass across different genotypes,^25^ with particular attention to water requirements for extraction and the concentration of extracted polyphenols. These findings offer valuable insights to further promote circular and green economy principles. The ambition to repurpose waste biomass for nutraceutical applications presents a key limitation in the choice of solvents because only food‐grade and environmentally friendly solvents can be considered for recovering bioactive molecules from artichoke waste biomass.^26^ In this context, significant variations in bioactive compounds among plant materials must be considered, as well as the eligibility of different extraction methods. These methods range from conventional techniques to innovative ones,^27^ and are generally based on the solvent's extracting power combined with the application of heat and/or mixing. Common methods include Soxhlet extraction, heat reflux, maceration, pulsed electric field, pressurized liquid, microwave‐assisted, ultrasonic‐assisted and subcritical fluid extraction.^28^, ^29^ With the increasing emphasis on sustainability, green and sustainable extraction techniques such as aqueous extraction are gaining attention^30^ because they efficiently recover key phenolic compounds in plant materials, including anthocyanins, tannins, terpenoids and flavonoids.^31^, ^32^

Therefore, the present study aimed to specifically explore the polyphenol composition and the potential bioactivity of these compounds when recovered from seven different artichoke varieties, at the same time as considering the plant agronomic features. Specifically, the functional properties of aqueous extracts (AEs) derived from the related by‐products (i.e. external bracts) were assayed. The bio‐functional evaluation of each AE focused on phenol profiling, radical scavenging and prebiotic activity.

## MATERIALS AND METHODS

### Chemicals and standards

Formic acid, water, and acetonitrile for spectrometry, gallic acid and 5‐*O*‐caffeoylquinic acid were purchased from Sigma‐Aldrich Co. (St Louis, MO, USA). Folin–Ciocalteu (F–C) reagent was obtained from Carlo Erba Reagent (Milano, Italy).

### Plant material

Seven different globe artichoke genotypes with different biological profiles were characterized morphologically and agronomically. Two out of these were selected as representative of commercial artichoke hybrids (Nunhems; BASF, Ludwigshafen, Germany): *Artemisa*, which meets the needs of the fresh and packed first‐range products markets, and *Capriccio*, for both the fresh and processing markets. The other five were selected from local Apulian varieties (*Brindisino*, *Locale di Mola Tardivo*, *Troianella* and *Violetto di Manfredonia*) and one Sardinian local variety (*Spinoso Sardo*), all of which meet the needs of the fresh and packed first‐range product markets. Plantlets were obtained from seed for hybrids and offshoots (vegetative propagation) for local varieties and transplanted into the open field in August 2022. The experimental field was set up in Ordona, in the north of the Apulia region (41°17′58″N, 15°37′52″E; altitude 127 m a.s.l.), with a planting density of 1.0 plants m^2^ (plants spaced 0.8 m apart, in rows 1.25 m apart). The climate in the area is subhumid climate according to De Martonne's climate classification with hot, dry summers and an average annual rainfall of approximately 490 mm, irregularly distributed throughout the year (approximately 40% in autumn). The annual temperature ranges between 3 and 33 °C, on average. The experimental design employed a randomized complete block layout with three replications for each treatment, and each experimental unit comprised 20 plants. The agronomic management of the trial consisted in applying a fertilization programme commonly used in the area for artichoke crops (200 kg N, 80 kg P_2_O_5_ and 100 kg K_2_O per ha). Drip irrigation was applied when 40% of the available water had been consumed in the soil layer most affected by the roots, replenishing 100% of the water lost. Plant protection was carried out according to the Integrated Pest Management protocol according to European Union directives.

### Morphological characteristics of genotype variety

Commercial head production, regardless of size, started in March 2023, with slight differences between the varieties studied. Heads were collected, weighed and measured for diameter and maximum length to the size suitable for packed first‐range, four per replica.

The colour of the outer bracts and the shape of the flower heads were recorded. These were based on the length/diameter ratio. For each variety, the production in heads per plant and per hectare was determined at the end of the cycle.

### Sample preparation

Globe artichokes belonging to hybrids and local varieties mentioned above were recovered in March 2023 as first harvest, upon reaching commercial maturity. Heads were harvested, bracts belonging to the most external layers (four rows) were recovered and labeled as by‐product samples. The outer bracts of each cultivar were washed and dried in an oven at 60 °C, until constant weight. The dry samples were blended using a laboratory knife mill, until reaching 0.5–1.0 mm and stored at 4 °C until use.

### AEs

Phenolic compounds were extracted from milled dry bracts (MDB), according to Mirpoor *et al*.,^33^ with slight modifications. Artichoke bract extracts were obtained using an automated laboratory extractor, Timatic Micro 0.5 (Technolab SrL, Spello, Perugia, Italy). A filter bag (porosity 50 μm) was filled in with 50 g of MDB and introduced in the extraction chamber (volume of 500 mL). Extraction was achieved using distilled water as solvent, according to the following parameters: biomass/solvent ratio 1:10 (w/v), room temperature, pressure between 5 and 9 bar (static phase 2 min; dynamic phase 2 min, 10 s piston stop), 10 cycles, five percolations. After the extraction process, the filter bag was removed from the chamber and squeezed to optimize the artichoke bract extract recovery. AEs were kept at 4 °C and immediately analyzed to determine total phenol content (TPC).

### TPC

AEs were evaluated for the TPC according to the F–C method,^34^ with slight modification as reported by Caponio *et al*.^35^ Briefly, to 980 μL of H_2_O Milli‐Q, 20 μL of appropriately diluted extract and 100 μL of F–C reagent were added. After 3 min, 800 μL of 7.5% Na_2_CO_3_ were added and then the sample was stored in the dark for 60 min. The absorbance was read at 720 nm using an Evolution 60s UV‐visible spectrophotometer (Thermo Fisher Scientific, Rodano, Italy). The results were expressed as mg of gallic acid equivalents (GAE) per litre of sample (mg GAE L^−1^). Each sample was analyzed in triplicate.

### Ultra‐high‐performance liquid chromatographic‐diode array (UHPLC‐DAD) analysis of the phenolic profile

As previously described,^36^ the identification and quantification of phenolic compounds was carried using a UHPLC Dionex Ultimate 3000 system (HPG 3200 RS binary pump, WPS‐3000 TRS autosampler, TCC‐3000 RS column thermostat compartment, DAD detector; Thermo Fischer Scientific, Germering, Germany). The separation of phenolic compounds was performed using an Acclaim 120 C18 column (120 Å 3 × 150 mm, 3 μm particle size; Thermo Fischer Scientific, Waltham, MA, USA) maintained at 30 °C. A mobile phase was used consisting of (A) water/formic acid (99.9:0.1, v/v) and (B) acetonitrile/formic acid (99.9:0.1, v/v) at a constant flow rate of 0.55 mL min^−1^. The gradient program of solvent A was: 0–1 min isocratic 94%; 1–26 min increase to 55%; 26–33 min decrease to 30%; 33–36 min isocratic 30%; 36–55 min increase to 94%. The detection was conducted at 330 nm and quantitative analysis was carried out using the external standard method based on a calibration curve obtained by injecting different concentrations of 5‐*O*‐caffeoylquinic acid ranging from 1000 mg L^−1^ to 7.8125 mg L^−1^ (*r* ^2^ = 0.9994–0.9997). The identification of the compounds was carried out by comparing the retention times and the spectral parameters of peaks with those of the standards according to standardized procedures.^37^ The results were expressed in mg of compound per liter of AE. The analysis was performed in triplicate.

### Scavenging activity

The antioxidant activity of the AE was assessed by measuring their ability to scavenge the radical 2,2‐diphenyl‐1‐picrilyhydrazil (DPPH•). The method used for determining the free radical scavenging activity against DPPH was adapted from the protocol of Kedare and Singh.^38^ Specifically, 50 μL of each sample was mixed with 950 μL of DPPH• solution (0.0031 g 100 mL^−1^ in ethanol). In addition to the samples, a vacuum control and a positive control were included. The vacuum control consisted of 50 μL of an ethanol‐water solution, whereas the positive control contained the synthetic antioxidant butylated hydroxytoluene as a reference (1 g L^−1^ in ethanol–water solution). After incubating the mixture in the dark for 30 min at 25 °C, the absorbance was measured at 517 nm in an Cary 60 spectrophotometer (Agilent, Cernusco, Italy). The free radical scavenging activity was calculated using:

$$\text{DPPH}•\text{scavenging activity}\left(\%\right)=\begin{array}{l} \left[\frac{\left(\text{blank absorbance}-\text{sample absorbance}\right)}{\text{blank absorbance}}\right]\\ \times 100 \end{array}$$

Data are expressed as the mean ± SD of three different experiments.

### Prebiotic activity

To assess the prebiotic activity of the extracts, bacterial strains of *Bifidobacterium* and lactobacilli (basonym *Lactobacillus*) were assayed. Specifically, these were *Bifidobacterium (B.) breve* 15A, *B. animalis* 13A, *Lactobacillus (L.) acidophilus* LA3, *L. delbrueckii* SP5, *Lacticaseibacillus (Lc.) casei* LC4P1, *Lc. casei* BGP93, *Lc. paracasei* 14A, *Lc. rhamnosus* LRB, *Lactiplantibacillus (Lp.) plantarum* 30N, *Lp. plantarum* LPAL, *Lp. plantarum* 8VEG3C, *Lp. plantarum* ONI17, *Lp. plantarum* ONI3, *Lp. plantarum* VEGI1, *Lp. plantarum* VEGII1, *Limosilactobacillus (Ls.) reuteri* ATCC23272 and Synbio100 (a mixture containing 1:1 *Lc. rhamnosus* IMC 501 and *Lc. paracasei* IMC502). An exception was made for Synbio100, which was supplied by SACCO Srl (Cadorago, Italy). All strains belonged to the Culture Collection of the Dept. of Soil, Plant and Food Science of University of Bari Aldo Moro.^39^

Modified (2 g L^−1^ of glucose, instead of 20 g L^−1^) de Man, Rogosa and Sharpe (m‐MRS) broth medium was prepared in lab as previously described^40^ and then supplemented with the different AEs to assess their potential prebiotic effects. Artichoke extracts were cold‐filtered (0.22 μm) and added (10%, v/v) to the sterile m‐MRS medium and the pH value (6.2 ± 0.2) was verified. As a control, m‐MRS broth without the addition of artichoke extract was used. Each strain was inoculated at a cell density of 5 × 10^7^ colony‐forming units mL^−1^ in 5 mL of m‐MRS broth, with or without the AE, and cultured at 37 °C for 24 h. Subsequently, cells were harvested by centrifugation (9000 × *g* for 10 min), washed twice with sterile potassium phosphate buffer at 50 mm (pH 7.0) and resuspended in an equal volume of saline solution (NaCl, 0.9 g L^−1^). The suspension was used for absorbance reading at 620 nm considering lectures of 0.250 corresponding to 10^8^ colony‐forming units mL^−1^ then verified by plated counts on MRS agar (Oxoid Ltd, Basingstoke, UK). The acidifying activity (pH values) resulting from probiotic fermentation was measured using an ultrabasic ub‐10 pH meter (Denver Instrument Company, Arvada, CO, USA) equipped with a food penetration probe.

### Statistical analysis

The results are expressed as the mean ± SD of artichoke extracts. Significant differences (*P* < 0.05) were determined by analysis of variance unidirectional (ANOVA), followed by Student Newman–Keuls (SNK) or Tukey's test for multiple comparisons. Statistical analysis was performed using Minitab (Minitab Inc., State College, PA, USA). Multivariate analysis accounted for the combination of data obtained from different assays, and according to data‐scaling (normalization; *z*‐scores), a principal component analysis (PCA) was performed and visualized using Prism, version 9.0.0 (GraphPad Software Inc., San Diego, CA, USA).

## RESULTS

### Morphological characteristics of heads

The artichoke varieties showed a significant variability in the examined morphological characters (Table 1). In particular, the weight of heads was significantly higher in the *Artemisa* and *Capriccio* hybrids, with values above 180 g compared to the local varieties for which values ranged from 113.4 g in *Violetto di Manfredonia* to 158.2 g in *Brindisino*. The length/diameter ratio confirmed the specificity of the head shapes of the different genotypes. Concerning this ratio, values higher than 1.2 were obtained for the hybrid *Capriccio* and the local varieties *Brindisino*, *Locale di Mola Tardivo* and *Spinoso Sardo*, characterized by cylindrical/conical heads of the *Catanese* artichoke type, whereas lower values were obtained for *Artemisa*, *Troianella* and *Violetto di Manfredonia*, characterized by spherical heads of the *Romanesco* type.

**Table 1:** Head morphological characteristics of seven genotypes of globe artichoke

Genotype	Fresh weight (g)	Length/diameter ratio	Color of outer bracts
Hybrids			
*Artemisa*	186.11 ± 2.72 a	1.03 ± 0.05 c	Dark purple – green hues
*Capriccio*	183.25 ± 3.04 a	1.38 ± 0.03 ab	Dark purple
Local varieties			
*Brindisino*	158.17 ± 1.91 b	1.25 ± 0.02 b	Green – purple hues
*Locale di Mola Tardivo*	120.78 ± 2.28 d	1.42 ± 0.06 a	Green – purple hues
*Spinoso Sardo*	145.57 ± 3.33 c	1.56 ± 0.02 a	Violet – brown hues
*Troianella*	141.90 ± 2.44 c	1.18 ± 0.03 b	Green – dark purple hues
*Violetto di Manfredonia*	113.44 ± 7.01 e	1.00 ± 0.01 c	Green – purple hues

All values are the mean ± SD of three replicates. Within the same column, different letters indicate a significant difference (*P* < 0.05; one‐way ANOVA and SNK test).

The color of the outer bracts varied from dark purple/violet with green to brown hues in the *Artemisa*, *Capriccio* and *Spinoso Sardo* genotypes, to green with purple hues in the others.

### Production yield of artichoke genotypes

In terms of production yield, the artichoke genotypes exhibited significant differences in the number of marketable heads. The hybrids demonstrated a clear superiority, yielding almost twice as many heads per plant compared to the local varieties, with 8.6 heads for *Capriccio* and 7.9 for *Artemisa* (Table 2). Among the local varieties, the most productive was *Spinoso Sardo*, with five heads per plant, followed by *Brindisino* and *Locale di Mola Tardivo*, which achieved similar results, specifically 4.1 and 4.3 heads per plant, respectively. The varieties *Troianella* (3.5) and *Violetto di Manfredonia* (2.3) recorded the lowest values. This clearly reflected to the production per hectare of marketable heads. Although the production of the two hybrids was significantly higher compared to local varieties, *Capriccio* showed a slightly higher, but not significant, value (15.7 t ha^−1^) than *Artemisa* (14.63 t ha^−1^). Among the local varieties, *Spinoso Sardo* showed the best production potential with a production of marketable heads per hectare of 7.33 t ha^−1^, followed by *Brindisino* (6.54 t ha^−1^), *Locale di Mola Tardivo* (5.23 t ha^−1^) and *Troianella* (4.97 t ha^−1^). The lowest value was found for *Violetto di Manfredonia* (2.65 t ha^−1^).

**Table 2:** Number and fresh weight of heads, by‐products fresh weight and yield of by‐products of the seven genotypes of *Cynara cardunculus* var. *scolymus* under study

Genotype	Heads per plant (*n*)	Heads yield (t/ha of fresh weight)	By‐products (estimated %)	By‐product (estimated % of dry matter)	By‐product (g/head of dry matter)	By‐product (t ha^−1^ dry matter)
Hybrids						
*Artemisa*	7.9 ± 0.31 a	14.63 ± 1,13 a	49	18	16.39 ± 1.00 a	1.29 ± 0.39 a
*Capriccio*	8.6 ± 0.57 a	15.70 ± 2,01 a	49	18	16.06 ± 2.50 a	1.38 ± 0.88 a
Local varieties						
*Brindisino*	4.1 ± 0.35 c	6.54 ± 2.86 bc	44	15	12.62 ± 4.10 c	0.52 ± 0.12 c
*Locale di Mola tardivo*	4.3 ± 0.32 c	5.23 ± 3.16 c	50	16	10.93 ± 1.53 d	0.47 ± 0.27 c
*Spinoso sardo*	5.0 ± 0.23 b	7.33 ± 1,75 b	48	15	12.70 ± 0.89 c	0.64 ± 0.24 bc
*Troianella*	3.5 ± 0.20 d	4.97 ± 1.96 c	53	15	13.50 ± 1.91 b	0.47 ± 0.11 c
*Violetto di Manfredonia*	2.3 ± 0.35 e	2.65 ± 0.96 d	49	17	9.94 ± 0.35 e	0.23 ± 0.09 d

All values are the mean ± SD of three replicates. Within the same column, different letters indicate a significant difference (*P* < 0.05; one‐way ANOVA and SNK test). Estimated entries were excluded from statistical comparison.

To recover biologically active substances from artichoke, the percentage of by‐products from heads was estimated at an average of 49%, without substantial variability among the different varieties. Of dry matter, the percentage of by‐products was highest in the two hybrids (18%), whereas it ranged between 15% and 17% in local varieties. The quantity of by‐products was directly influenced by the average weight of heads reported in Table 1. A value of by‐products close to 16 g (dry matter) per head was observed in the two hybrids, the only varieties exceeding 180 g in head weight. By contrast, the yield of by‐products of local varieties ranged from 13.5 g (*Troianella*) to 9.94 g (*Violetto di Manfredonia*).

Consequently, an estimation of the by‐products obtainable from the production per hectare of commercial heads highlighted a clear advantage for the hybrids, yielding 1.29 t ha^−1^ for *Artemisa* and 1.38 t ha^−1^ for *Capriccio*. Among the local varieties, the highest by‐product yield was observed in *Spinoso Sardo* (0.64 t ha^−1^), whereas the lowest was recorded for *Violetto di Manfredonia* (0.23 t ha^−1^). The remaining local varieties produced by‐products ranging between 0.47 t ha^−1^ (*Troianella*) and 0.52 t ha^−1^ (*Brindisino*) (Table 2).

### Quantitative analysis of phenolic compounds of aqueous extracts

The AEs were analyzed by UHPLC‐DAD. The quantified phenolic compounds are reported in Table 3. Overall, the observed results highlighted that 5‐*O*‐caffeoylquinic acid and 3,4‐di‐*O*‐caffeoylquinic acid are the most abundant hydroxycinnamates in AEs, whereas the main flavonoids are apigenin‐7‐*O*‐rutinoside and luteolin‐7‐*O*‐glucoside. In detail, on average, *Violetto di Manfredonia* exhibited the highest values, significantly differing from the other samples. Particularly noteworthy were the contributions of 5‐*O*‐caffeoylquinic acid (16.39 mg L^−1^) and 3,5‐di‐*O*‐caffeoylquinic acid (11.96 mg L^−1^), followed by apigenin‐7‐*O*‐rutinoside and 3,4‐di‐*O*‐caffeoylquinic acid, with concentrations of 5.8 and 4.99 mg L^−1^, respectively. An additional sample rich in phenolic compounds was *Locale di Mola Tardivo* (22.67 mg L^−1^), which significantly differed compared to all other extracts. No significant difference was found in the sum of phenolic compounds between *Brindisino* (15.02 mg L^−1^ ± 0.84), *Capriccio* (16.07 mg L^−1^ ± 1.35) and *Spinoso Sardo* (13.62 mg L^−1^ ± 1.28). In terms of overall phenol concentration, *Troianella* showed the lowest value (2.97 mg L^−1^) without significant differences compared to *Artemisa* (7.04 mg L^−1^).

**Table 3:** Quantified content (mg L^−1^, mean ± SD) by the UHPLC‐DAD analysis of the main phenolic compounds of artichoke aqueous extracts (AE) from the seven different plant varieties

Compound	Artemisa	Brindisino	Capriccio	Locale di Mola Tardivo	Spinoso Sardo	Troianella	Violetto di Manfredonia
5‐*O*‐caffeoylquinic acid	2.55±0.08 c	0.78±0.05 d	0.33±0.01 d	0.91±0.55 d	7.16±0.69 b	0.07±0.01 d	16.39±0.21 a
3,4‐di‐*O*‐caffeoylquinic acid	1.48±0.07 e	8.39±0.23 c	10.53±0.26 b	14.61±0.36 a	2.50±0.05 e	1.42±0.09 e	4.99±0.9 d
3,5‐di‐*O*‐caffeoylquinic acid	1.78±0.01 c	0.47±0.39 d	1.6±0.22 cd	4.04±0.64 b	1.99±0.1 c	0.86±0.16 cd	11.96±0.27 a
Luteolin‐7‐*O*‐rutinoside	0.69±0.05 a	0.52±0.08 a	0.29±0.05 a	0.19±0.26 a	0.22±0.31 a	0.2±0.03 a	0.4±0 a
Luteolin‐7‐*O*‐glucoside	0.11±0.02 d	2.73±0.11 a	1.45±0.27 b	0.96±0.36 bc	0.46±0.06 cd	0.17±0.01 d	0.09±0.0 d
Luteolin‐7‐*O*‐glucorinide	0.17±0.01 de	0.85±0.04 a	0.75±0.17 ab	0.39±0.05 bcd	0.59±0.07 abc	0.06±0.01 de	0.3±0.18 cd
Apigenin‐7‐*O*‐rutinoside	0.13±0.03 b	0.83±0.09 b	0.61±0.2 b	0.93±0.19 b	0.6±0.01 b	0.12±0.01 b	5.8±1.01 a
Apigenin‐7‐*O*‐glucoronide	0.13±0.03 ab	0.44±0.05 ab	0.51±0.17 ab	0.64±0.33 a	0.10±0.01 ab	0.07±0.01 b	0.56±0.03 ab
Total	7.04±0.12 d	15.02±0.84 c	16.07±1.35 c	22.67±1.11 b	13.62±1.28 c	2.97±0.29 d	40.49±1.78 a

All values are the mean ±SD of three replicate measurements. Different letters indicate a significant difference (*P* < 0.05; one‐way ANOVA).

### Aqueous extract and polyphenol yield

Considering the merging of data related to the production yield of each variety and the related by‐product biomass (Table 2), the total polyphenol content in AE (Table 4) was calculated as the relative yield per hectare. The highest volume of AE was found for the hybrid varieties that, being characterized by the highest production potential, had an estimated value of 13 760 L per hectare (*Capriccio*) and 12 880 L per hectare (*Artemisa*), whereas local varieties yielded a volume ranging between 2190 L per hectare (*Violetto di Mola*) and 5330 L per hectare (*Spinoso Sardo*).

**Table 4:** Estimation of artichoke by‐products tons, liters of extract and total polyphenols per hectare of crops of the seven genotypes of *Cynara cardunculus* var. *scolymus* under study

Genotype	Total artichoke AE (1000 L ha^−1^)	Total phenol yield (g ha^−1^)
Hybrids		
*Artemisa*	12.88 ± 2.41 a	90.68 ± 18.07 b
*Capriccio*	13.76 ± 4.16 a	221.12 ± 12.32 a
Local varieties		
*Brindisino*	4.35 ± 0.11 c	65.27 ± 22.51 c
*Locale di Mola Tardivo*	4.21 ± 0.95 c	95.40 ± 14.31 b
*Spinoso Sardo*	5.33 ± 1.05 bc	72.55 ± 15.84 c
*Troianella*	3.94 ± 0.87 c	11.70 ± 22.17 d
*Violetto di Manfredonia*	2.19 ± 0.36 d	88.66 ± 13.26 bc

AE, aqueous extract. All values are the mean ±SD of three replicates. Different letters indicate a significant difference (*P* < 0.05; one‐way ANOVA).

The total polyphenols, expressed in mg L^−1^, and the volume of each AE per hectare were used to estimate the total polyphenol yield in grams per hectare. The highest value was recorded for the hybrid *Capriccio* (221.12 g ha^−1^), which was more than two‐fold higher than the other hybrid variety *Artemisa* (90.68 g ha^−1^).

Among the local varieties, *Locale di Mola Tardivo* had the highest value (95.40 g ha^−1^), followed by *Violetto di Manfredonia* (88.66 g ha^−1^), *Spinoso Sardo* (72.55 g ha^−1^) and *Brindisino* (65.27 g ha^−1^), whereas *Troianella* showed the lowest (11.70 g ha^−1^).

### TPC

**Figure 1:**
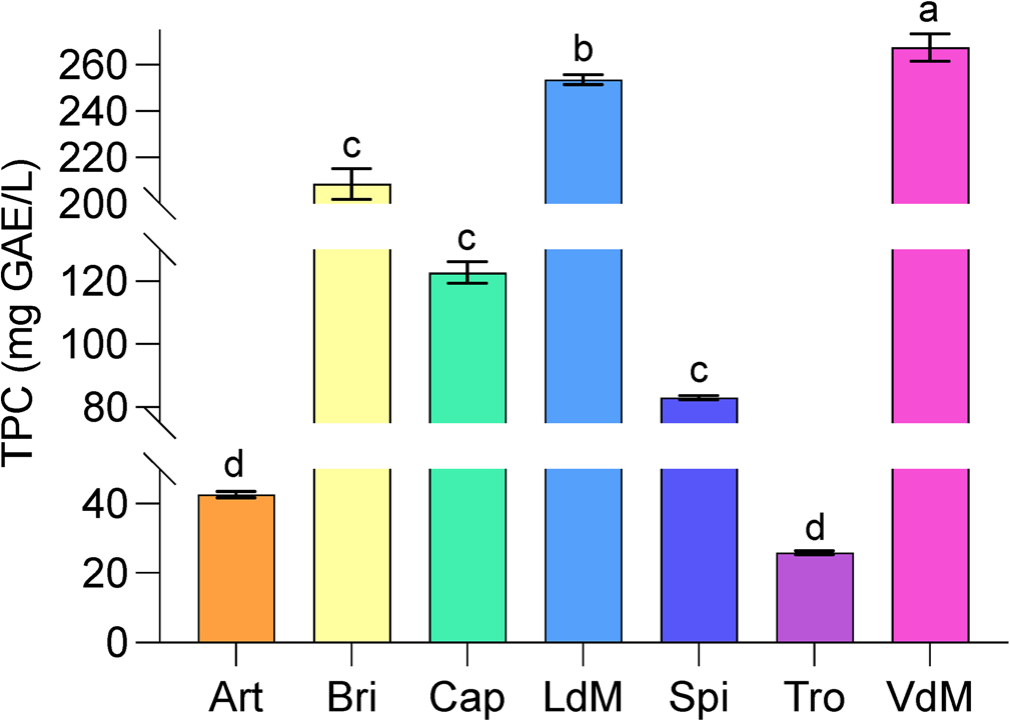
Total phenol content (TPC) of artichoke AE from seven different verities of artichoke, specifically: Artemisa, Art; Brindisino, Bri; Capriccio, Cap; Locale di Mola Tardivo, LdM; Spinoso Sardo, Spi; Troianella, Tro; Violetto di Manfredonia, VdM. Abbreviations: GAE, gallic acid equivalent; TPC; total phenol content. The values represent means of triplicates ±SD. On staked bars, different letters indicate a significant difference (*P* < 0.05; one‐way ANOVA).

The extracts were also analyzed for their TPC using the F–C colorimetric assay, and the results are presented in Fig. 1. Consistent with the UHPLC‐DAD findings, significant differences in TPC values were observed among the AEs of the different cultivars. Specifically, *Violetto di Manfredonia* exhibited the highest TPC value, reaching 267.45 ± 5.98 mg GAE L^−1^, which significantly differed from all other extracts. This was followed by *Locale di Mola Tardivo*, with a value of 253.5 ± 2.11 mg GAE L^−1^. These samples showed significant differences compared to *Brindisino* and Capriccio samples, which did not differ significantly from each other, with values of 208.5 ± 6.57 mg GAE L^−1^ and 122.7 ± 3.3 mg GAE L^−1^, respectively. The remaining samples (*Spinoso Sardo*, *Artemisa* and particularly *Troianella*) exhibited the lowest TPC values compared to others.

### Scavenging activity

**Figure 2:**
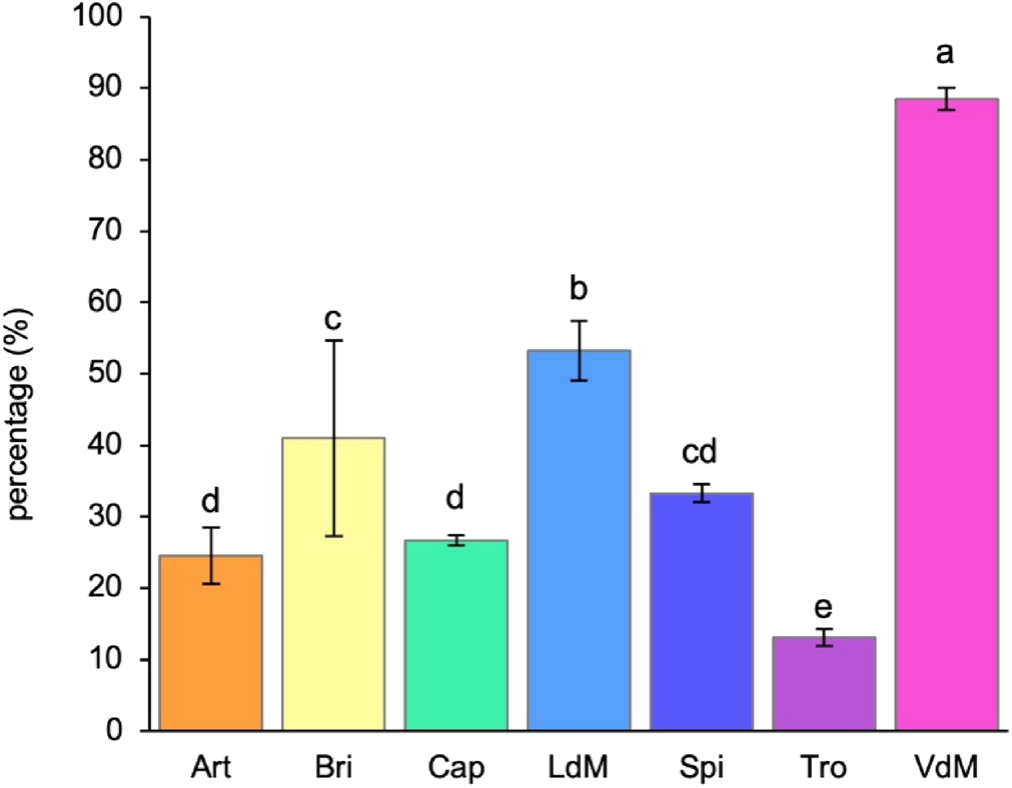
Scavenging activity (%) against the DPPH• free radical assayed by using artichoke AE from seven different verities of artichoke, specifically: Artemisa, Art; Brindisino, Bri; Capriccio, Cap; Locale di Mola Tardivo, LdM; Spinoso Sardo, Spi; Troianella, Tro; Violetto di Manfredonia, VdM. On staked bars, different letters indicate a significant difference (*P* < 0.05; one‐way ANOVA).

The resulting bioactivity of total phenols was studied as radical scavenging activity (Fig. 2). From the comparison between AEs of artichoke varieties, it was observed as *Violetto di Manfredonia* (88.4% ± 1.5) and *Troianella* (13.1% ± 1.2) reported the highest and lowest values, respectively, and these significantly differed compared to the other five samples. Moreover, *Locale di Mola Tardivo* (53.2% ± 4.2) significantly differed compared to all other extracts. No difference was found between extracts of *Brindisino* (41% ± 13.7) and *Spinoso Sardo* (33.3% ± 1.3) but, although the former significantly differed compared to both extracts from hybrids (*Artemisa* and *Capriccio*; *P* = 0.003 and 0.008, respectively), the same was not between *Spinoso Sardo* and hybrid varieties.

### Prebiotic activity

**Figure 3:**
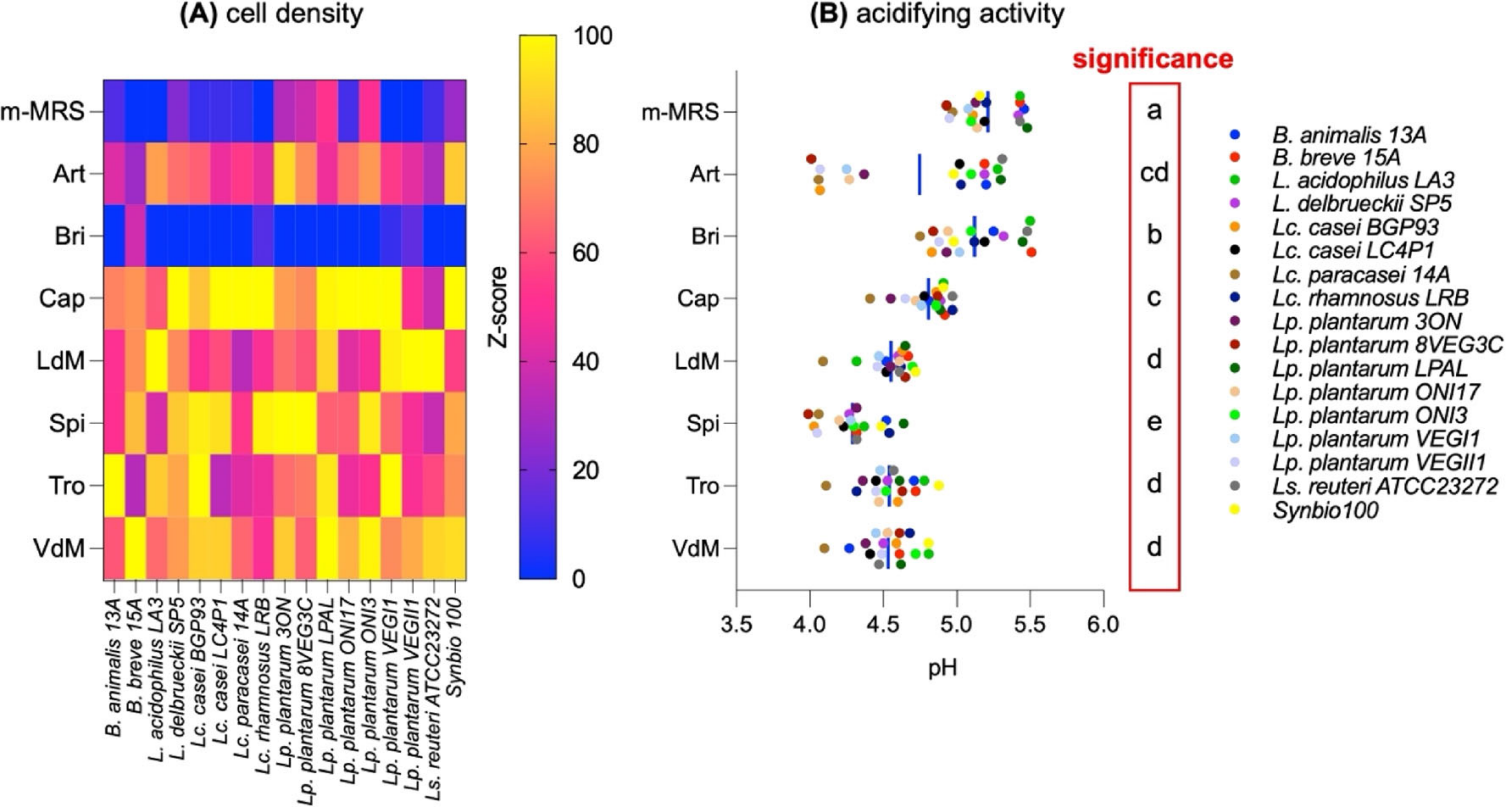
Prebiotic activity of artichoke AE from by‐products (external bracts) of seven different varieties according to (A) probiotic cell density after 24h of fermentation and (B) the related acidifying activity. The m‐MRS medium was used as control, whereas AE were added at 10% in m‐MRS. Into the column ‘significance’ of (B), different letters indicate a significant difference (*P* < 0.05; one‐way ANOVA).

The prebiotic activity gained by the addition of artichoke AE during microbe culturing conditions was assessed on 17 different probiotics, including two bifidobacterial strains, 14 different lactobacilli (basonym *Lactobacillus*) and one commercial probiotic mixture encompassing two different species of the *Lacticaseibacillus* genus. After 24 h of fermentation, this was inspected in terms of microbial cell density (Fig. 3A), as well as probiotic acidifying activity (Fig. 3B). Both the control medium (m‐MRS) and the *Brindisino‐*extract reported the lowest scores for most of the tested strains. By contrast, the *Capriccio* extract displayed the highest capability to harbor probiotic cell density, followed by *Spinoso Sardo* and *Violetto di Manfredonia*. Intermediate values were found in *Troianella*, *Locale di Mola Tardivo* and *Artemisa* extracts, with *Artemisa* exhibiting the lowest average among these three samples. Almost for the totality, results from pH inspection were in line with data from cell density. Indeed, m‐MRS displayed the highest scores of pH, reaching significance compared to all media supplemented with artichoke AE. Moreover, the pH in *Brindisino* samples, showing mean values of 5.1 ± 0.26 (minimum to maximum: 4.8–5.5), was significantly higher than the other six artichoke AEs. No difference was found between *Capriccio* (4.8 ± 0.15) and *Artemisa* (4.7 ± 0.52). However, although the latter did not show differences compared to *Locale di Mola Tardivo* (4.6 ± 0.16), *Troianella* (4.5 ± 0.18) and *Violetto di Manfredonia* (4.5 ± 0.18), the pH values in *Capriccio* were significantly higher than those found in *Locale di Mola Tardivo*, *Troianella* and *Violetto di Manfredonia*. Lastly, the lowest pH values, 4.3 ± 0.19, minimum to maximum: 4.0–4.6, were found in *Spinoso Sardo* samples.

### Multivariate study

**Figure 4:**
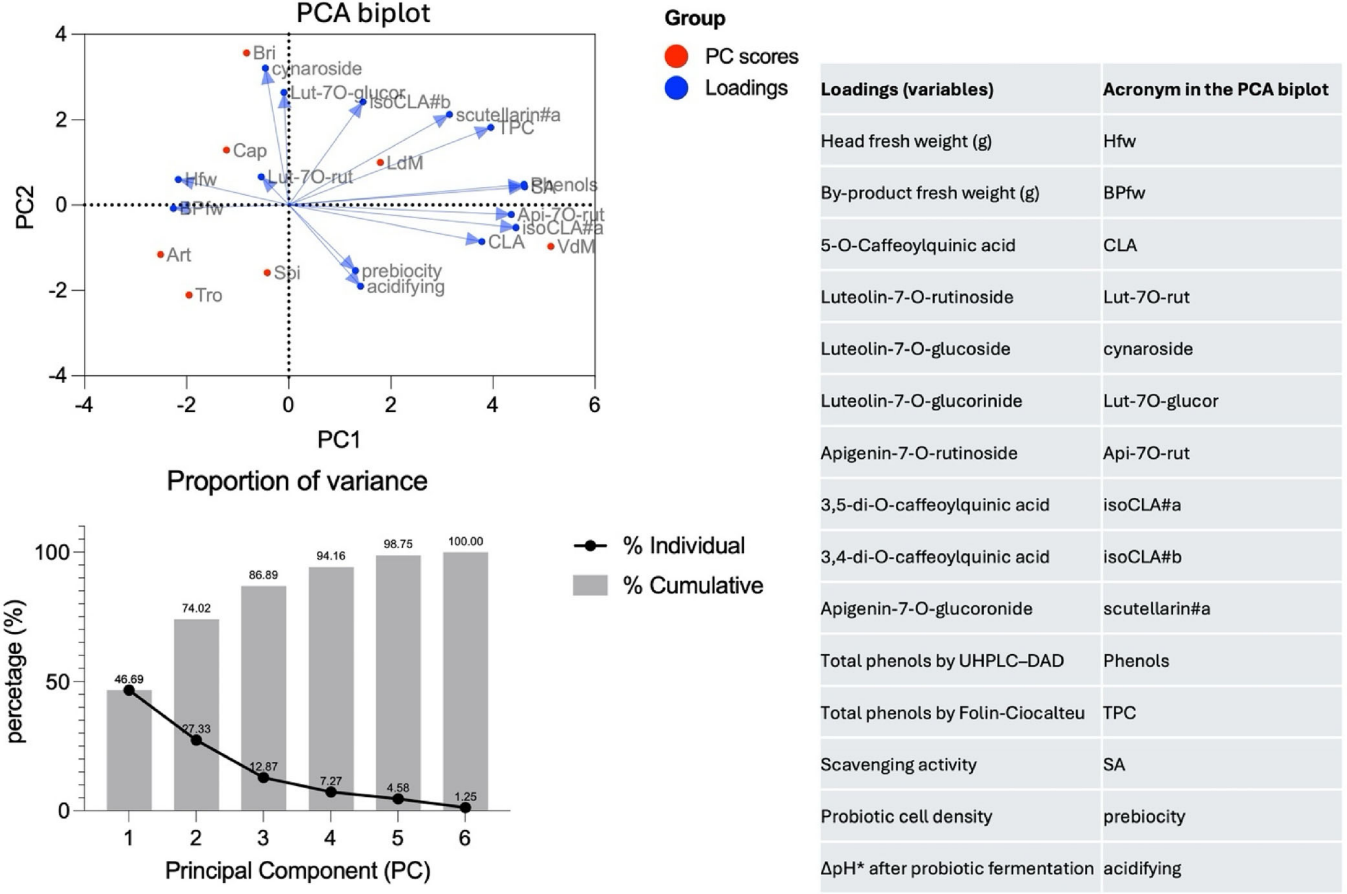
Principal component analysis (PCA) biplot and PC contribution to total variance. Both prebioticity and acidifying were calculated as the mean value of the 17 probiotics‐based experiments. ΔpH* considered the difference between the baseline value (6.2 ± 0.2) and the pH assessed according to probiotics fermentation. The varieties under study were: Artemisa, Art; Brindisino, Bri; Capriccio, Cap; Locale di Mola Tardivo, LdM; Spinoso Sardo, Spi; Troianella, Tro; Violetto di Manfredonia, VdM.

To obtain an overall description of the interaction between the obtained data, a multivariate approach was used, and results were visualized in a PCA biplot (Fig. 4). The firsts two principal components (PC1 and PC2) explained 74.02% of the total variance. PC1 was useful for differentiating samples of *Artemisa*, *Capriccio* and *Troianella* based on high values of both artichoke head and by‐products weights according to negative PC1 scores to those samples (i.e. *Locale di Mola Tardivo* and *Violetto di Manfredonia*) showing high values of total phenols measured by both F–C and UHPLC‐DAD, high concentration of specific phenolic compounds (i.e. CLA, iso‐CLA type‐a, apigenin‐7‐*O*‐rutinoside and scutellarin type‐a) and high radical scavenging activity, as supported by positive PC1 scores. PC2, instead allowed discrimination of samples *Artemisa*, *Spinoso Sardo*, *Troianella* and *Violetto di Manfredonia* showing the highest prebiotic activity, in terms of probiotics cell density after fermentation, and acidification values according to negative PC2 scores from samples *Brindisino*, *Capriccio* and *Locale di Mola Tardivo* showing the highest concentration specific phenol compounds (i.e. cynaroside, Lut‐7O‐glucorinide and iso‐CLA type‐b).

## DISCUSSION

Research concerning the bioactivity of artichoke compounds has suggested several potential health benefits.^41^ The abundant bioactive constituents found in artichoke by‐products, including leaves, outer bracts and stems, have been extensively documented.^4^, ^5^, ^8^, ^10^, ^12^, ^14^, ^20^, ^21^, ^42^, ^43^, ^44^, ^45^, ^46^ These components are characterized by low‐fat content and elevated levels of vitamins (C, K and some belonging to the B‐group), insoluble fibers, inulin and minerals (K, Na and P).^12^ Furthermore, artichoke by‐products exhibit a notable presence of various phenolic compounds and derivatives (i.e. flavonoids and hydroxycinnamic acids).^4^ Therefore, with the increasing market demands for artichoke‐based nutraceutical/pharmaceutical products, cultivating and valorizing globe artichoke varieties with a rich profile in bioactive compounds is of great interest to reduce the risks of diseases linked to modern lifestyle through the up‐cycling of bio‐functional ingredients rich in antioxidant and anti‐inflammatory activity. To our best knowledge, this work analyzed, for the first time, seven different varieties (two hybrids and five locals) agronomically and in terms of bioactivity of AE from the related by‐products (i.e. outer bracts).

As suggested in previous studies,^47^, ^48^ the hybrids investigated in the present study (*Artemisa* and *Capriccio*) exhibited the highest number of heads per hectare and the greatest head fresh weight as a result of selective breeding aimed at maximizing the production. However, this was insufficient to reduce the substantial biomass of head by‐products because both had also the highest yield of by‐products, constituting approximately 50% of the total fresh weight. The by‐product biomass tends to vary because of the different production potentials of hybrids compared to the local varieties. On average, the potential availability of by‐products from hybrids, harvested from each hectare, is expected to be more than one ton compared to the half‐a‐ton of local varieties. However, the availability of by‐products has not yet found an exact place in the agro‐industry, despite the numerous possibilities of their valorization as functional ingredients.^49^, ^50^ On the other hand, recognizing and utilizing artichoke by‐products as food supplements and functional ingredients could enhance the crop's profitability, providing significant benefits to the agricultural sector. This aligned with the potential yield of total polyphenols that can be extracted from the by‐products obtained from processing of artichoke and their commercial value. Noteworthy, the AE from *Violetto di Manfredonia* contained the highest concentration of 5‐*O*‐caffeoylquinic acid, namely the so‐called chlorogenic acid (CLA). Widely present in various plants and fruits, CLA is a prominent constituent of AEs and it is particularly recognized for its antioxidant properties contributing significantly to overall health benefits.^42^ Oxidative stress is often associated with numerous human diseases, such as chronic inflammation,^51^ Alzheimer's disease,^52^ type 2 diabetes mellitus,^53^ celiac disease^54^ or skin disorders.^55^ Therefore, the application of these AEs takes its place for a hypothetical treatment of various diseases. Consistent with previous studies showing how AEs can prevent reactive oxygen species formation and improve antioxidant status *in vitro* ^43^, ^56^ and in animals,^44^, ^57^, ^58^ the AE from the local variety *Violetto di Manfredonia*, followed by *Locale di Mola Tardivo*, reported the highest TPC leading to notable antioxidant activity, as evaluated by the radical scavenging assay. Indeed, various clinical trials have previously revealed slight improvements in antioxidant status in humans after oral intake of artichoke extracts, providing health outcomes even in terms of improved levels of several serum enzymes, such as malondialdehyde, oxidized‐low density lipoprotein, glutathione peroxidase and superoxide dismutase.^59^, ^60^ In line with these considerations, both *Violetto di Manfredonia* and *Locale di Mola Tardivo* can be potentially flagged as the most promising varieties to design further studies aimed at improving health in different patients.

The UHPLC‐DAD profiling of AE from the seven *C. scolymus* varieties allowed the identification and quantification of different compounds belonging to flavonoid and caffeoylquinic acid classes. This agrees with previous data in the literature with respect to CLA, di‐caffeoylquinic acids and flavonoids that have been previously reported in artichoke extracts.^61^, ^62^ Flavonoids constitute a well‐represented group of specialized metabolites in artichoke, with apigenin, luteolin, apigenin‐7‐*O*‐rutinoside, apigenin‐7‐*O*‐glucoside (anthemoside), apigenin‐7‐*O*‐glucuronide (scutellarin A), luteolin‐7‐*O*‐rutinoside (scolymoside), luteolin‐7‐*O*‐glucoside (cynaroside) and luteolin‐7‐*O*‐glucuronide formerly reported.^61^, ^62^

However, it should be noted that the profile of bioactive components into plant‐based extracts can be influenced by various factors, including the type (i.e. variety) of artichoke used, growth conditions and extraction techniques. As the result of the interaction between all these conditions, the composition of phenolic compounds in artichoke extracts can display a large variability. Negro *et al*.^21^ previously investigated these differences as the result of qualitative and quantitative assessment of the phenolic profiling of six artichoke varieties. The analysis included three bract orders, from external to the inner section, different parts of the plant and different developmental stages of the plant. Fourteen compounds that belong to the hydroxycinnamate and flavone groups were identified using an HPLC‐mass spectrometry (MS) based approach. In line with our assays, the TPC varied significantly between genotypes (*P* < 0.05), but Negro *et al*.,^21^ suggested how this was more significantly between plant parts (*P* < 0.01). Also, in studies by Pandino *et al*.^63^ and Fratianni *et al*.,^20^ the phenolic composition differed significantly between cultivars and within different parts of the artichoke head. Moreover, how certain compounds have a tendency to accumulate selectively in specific parts of the plant was evaluated, with this increasing from external to internal parts, approximately 1700 (outer bracts) to 4500 (receptacle) mg kg^−1^ of dry matter, and in specific cultivars.^63^ Interestingly, the hydroxycinnamic acid content was either absent or very low in the outer bracts (443 mg kg^−1^ dry matter), whereas the edible portion of the receptacle contained the highest concentration of these compounds (average 1473 mg/kg^−1^ dry matter).^63^

Besides the instrument (e.g. liquid chromatography‐MS, gas chromatography‐MS or NMR) used for the artichoke by‐products profiling and compound identification, the polyphenol content also varies according to the extraction technique used.^46^ On average, CLA (5‐*O*‐caffeoylquinic acid) is the most abundant single substance (approximately 39%), followed by other caffeic acid derivatives, such as 1,5‐*O*‐dicaffeoylquinic acid (approximately 21%) and 3,4‐*O*‐dicaffeoylquinic acid (approximately 11%).^4^ CLA is the most widely recognized phenolic acid and, as such, is used as a quality control indicator in the artichoke herbs trade. The European Pharmacopoeia (Ph. Eur.) stipulates that artichoke leaves used as herbal drugs must have at least 0.7% CLA.^64^ However, the defined method, which involves heat reflux and two cycles of heating the sample in methanol up to 70 °C for 1 h each, is time‐consuming and requires a substantial quantity of organic solvents. For this reason, a field of research explored how different extraction strategies can influence this result. Stumpf *et al*.^65^ investigated whether the ultrasound‐assisted extraction (UAE) could replace the conventional Ph. Eur. procedure. Stumpf *et al*.^65^ considered concentrations of specific phenolic compounds and their antioxidant properties, examining additional factors such as the choice of the solvent. They compared the phenolic compound yield from methanol extraction (ME) to that from hot‐water extraction (HWE). They observed differences in the concentration of CLA, ranging from 0.8% to 3.38% dry matter in ME. The CLA and antioxidant capacity of the ME and UAE extracts were comparable, whereas cynaroside (−16%), total flavonoids (−11%), and TPC (−2.5%) of the UAE extracts were significantly lower than ME. On average, concentrations of caffeoylquinic acids were 4.5 and 5.1 times higher than flavonoid concentrations for ME and UAE extracts, respectively, whereas HWE extracts showed a significant decrease in extraction efficiency of CLA (−9.45%), total caffeoylquinic acids (−25%), cynaroside (−33.6%), total flavonoids (−22.2%) and TPC (−9.7%). In comparison to ME, chromatograms of HWE revealed two additional peaks of caffeoylquinic acid derivatives.^65^ Similarly, Garcia‐Castello *et al*.^66^ optimized the extraction method from artichoke wastes using HWE (89 °C) or hot water–ethanol mixtures (81 °C). Although HWE proved optimal for simultaneous inulin and polyphenol extraction, water–ethanol (EtOH = 22.4%) extractions produced the best results in terms of TPC (90%) and scavenging activity (38%), despite the lower inulin extracted. In line with previous works, we focused on recovering functional and bioactive compounds from artichoke by‐products using water as the solvent and verifying their prebiotic activity. However, to meet the sustainability principles, we here performed the extraction phase at room temperature. In terms of prebiotic activity, the best performance was exhibited by *Spinoso Sardo* AE. However, as shown by the PCA, AEs from *Violetto di Manfredonia*, *Locale di Mola Tardivo* and *Troianella* also demonstrated a considerable prebiotic activity. Thus, both *Locale di Mola Tardivo* and *Violetto di Manfredonia* samples were noteworthy for their functional and bioactive properties.

Previous studies have highlighted that artichoke bracts can contain bioactive compounds such as fructooligosaccharides,^4^, ^67^ and fructooligosaccharides exert a crucial role in the diet by selectively stimulating the growth and/or activity of beneficial microorganisms in the colon.^67^ The bifidogenic effect of artichoke extracts was previously evaluated using *in vitro* gut models based on the Simulator of Human Intestinal Microbial Ecosystem (SHIME), and assessing the production of health‐related microbial metabolites potentially attributable to inulin fermentation.^68^ Similarly, beneficial bacteria, including lactic acid bacteria and bifidobacteria, were grown using *in vitro* fermentation of artichoke by‐products with human fecal microbiota,^69^ and the results confirmed the suitability of artichoke extracts to increase densities of the pattern of health promoting bacteria. The same study also revealed that pathogenic bacteria, such as coliforms and sulphite‐reducing clostridia, were significantly reduced according to the artichoke substrate fermentation.^69^ In terms of bacterial metabolism assessment, the artichoke by‐products led also to the highest levels of short‐chain fatty acids because more than half of the artichoke substrate was utilized, confirming its prebiotic potential.^69^ However, it should be noted that our protocol was not optimized for the extraction of saccharides from bracts, which, on average, requires a water temperature higher than 60 °C.^66^, ^70^, ^71^ For this reason, the significant contribution to the presumptive prebiotic activity observed in our assays is probably attributable to the polyphenol content in the AEs because this was previously demonstrated by different studies.^72^, ^73^, ^74^ Furthermore, the effective prebiotic activity needs to be further and specifically evaluated considering that previous studies sustained that artichoke extracts exhibit antimicrobial effects against both Gram‐positive and Gram‐negative bacteria,^45^, ^75^ suggesting that the prebiotic‐to‐antimicrobial effect may be influenced under a dose‐dependent manner to a large spectrum of microbes. Whatever the prebiotic activity is, recent advances in field of circular and green economy in the agri‐food chain reported promising results supported by the fortification of staple foods, such as breads^56^, ^76^, ^77^, ^78^ or pasta,^79^, ^80^, ^81^ with solid waste of extracts from artichoke by‐products.

In conclusion, out of the seven *C. scolymus* varieties evaluated in this preliminary study, two varieties (*Violetto di Manfredonia* and *Locale di Mola Tardivo*) emerged as rich sources of bioactive and functional metabolites with potential antioxidant and prebiotic activity. This could provide a first insight into their health benefits and medicinal uses as bio‐functional ingredients to be added in fortified innovative foods. Consequently, artichoke bracts can be regarded as agricultural by‐products with high potential added value, opening new perspectives for their future exploitation and valorization. However, further studies and potential clinical trials are necessary to confirm the observed bio‐functional properties of the most promising local *C. scolymus* varieties *Violetto di Manfredonia* and *Locale di Mola Tardivo*.

## CONFLICTS OF INTEREST

The authors declare that they have no conflicts of interest.

## AUTHOR CONTRIBUTIONS

GRC, MV and LS were responsible for formal analysis. GRC, MV and LS were responsible for data curation. GRC, MV and LS were responsible for writing the original draft. GRC and MV were responsible for visualization. LS, AA, RS and GD were responsible for investigations. CR, GD and GDM were responsible for reviewing and editing. MDA and GDM were responsible for supervision. MDA and GDM were responsible for project administration. MDA was responsible for funding acquisition.

## Data Availability

The data that support the findings of this study are available from the corresponding author upon reasonable request.
